# Ages at peak height velocity in male soccer players 11–16 years: relationships with skeletal age and comparisons among longitudinal studies

**DOI:** 10.5114/biolsport.2024.127385

**Published:** 2023-07-19

**Authors:** Robert M. Malina, Miroslav Králík, Sławomir M. Kozieł, Sean P. Cumming, Jan M. Konarski, Paulo Sousa-e-Silva, Diogo V. Martinho, Antonio J. Figueiredo, Manuel J. Coelho-e-Silva

**Affiliations:** 1 University of Texas at Austin, Department of Kinesiology and Health Education; 2 University of Louisville, School of Public Health and Information Sciences, Louisville, Kentucky, USA; 3 Masaryk University, Faculty of Science, Department of Anthropology, Brno, Czech Republic; 4 Polish Academy of Sciences, Hirszfeld Institute of Immunology and Experimental Therapy, Department of Anthropology, Wrocław, Poland; 5 Bath University, Department of Health, Bath, UK; 6 Poznań University of Physical Education, Theory of Sports Department, Poznań, Poland; 7 University of Coimbra, FCDEF, Coimbra, Portugal; 8 University of Coimbra, CIDAF (uid/dtp/042143/2020), Coimbra, Portugal

**Keywords:** Adolescent Spurt, Youth Athletes, Maturity Timing, Maturity Status, Talent Identification

## Abstract

Estimated ages at take-off (TO) and at peak height velocity (PHV) based on two models and maturity status based upon age at PHV and skeletal age (SA) were compared in a longitudinal sample of male soccer players. In addition, estimated ages at PHV in 13 longitudinal samples of soccer players were compared. The longitudinal height records of 58 players of European ancestry, measured annually on four or five occasions between 11 and 16 years, were modeled with Superimposition by Translation and Rotation (SITAR) and Functional Principal Component Analysis (FPCA) to estimate ages at TO and PHV. SAs were assessed with the Fels method. Ages at PHV in 13 longitudinal samples of soccer players (Europe 7, Japan 6) were evaluated with meta-analysis. Estimated ages at TO, 11.2 ± 0.8 (SITAR) and 11.0 ± 0.8 (FCPA) years, and at PHV, 13.6 ± 0.9 (SITAR) and 13.7 ± 0.0 (FCPA) years, were similar. An earlier age at PHV was associated with advanced skeletal maturity status (rho = -0.77 at ~14 years). Ages at PHV among European players indicated a north (later) – south (earlier) gradient, and were later than ages at PHV among Japanese players. In summary, ages at TO and PHV were similar with SITAR and FPCA, and ages at PHV were most strongly correlated with SA at ~14 years. Mean ages at PHV showed a north-south gradient among European samples, and were later compared to Japanese samples.

## INTRODUCTION

Acceleration in the rate of growth in height in late childhood/early adolescence marks the onset or take-off (TO) of the adolescent growth spurt. The rate of growth accelerates until it reaches a peak (peak height velocity, PHV) and then decelerates until growth in height ceases in late adolescence or young adulthood. Ages at TO and at PHV and other parameters of the adolescent spurt are estimated from longitudinal height records. Early estimates were based on graphic plots of heights for individuals or on estimated increments in height between measurements. The development of mathematical models for evaluating longitudinal height records subsequently facilitated estimates of the parameters. Most models provide estimates of age at PHV (years), PHV (cm/year) and height at PHV (cm), while some also provide estimates of age, velocity of growth and height at TO, and of adult height [1–5]. Nevertheless, the procedures provide a convenient means for comparing individual and/or group differences in parameters of the adolescent spurt in height [6].

Longitudinal studies of parameters of the growth spurt are largely limited to samples from Europe, North America and Japan, and to a lesser extent Latin America. Estimated ages at PHV among youth from the different regions overlap, although estimates for Japanese youth tend to be somewhat earlier. Recent studies of U.S. youth also indicate overlap in mean ages at PHV among ethnic groups, though estimates for American Black youth tend to be at the early end of the distribution. Corresponding data for youth in other geographic areas are limited [6].

Ages at PHV based on longitudinal samples of youth participating in different sports, in contrast, are not extensive [7, 8]. This is a function of difficulties inherent in longitudinal studies *per se*, and also the selectivity of sport, differential persistence in a sport (drop out) and associated factors, e.g., injury, changing interests, and changes in teams/clubs, among others. Nevertheless, coaches and trainers are increasingly interested in monitoring growth in heights and weights of youth players over relatively short intervals in an effort to individualize training and to reduce the risk of injury during the adolescent growth spurt [9, 10]. As such, variation in the timing and intensity of growth in height at TO and PHV among youth athletes is important.

In the context of the preceding, the purposes of this study are threefold: first, to compare two methods for estimating parameters of the adolescent growth spurt in a longitudinal sample of male soccer players 11–16 years of age; second, to evaluate maturity classifications based on age at PHV, an indicator of maturity timing, and on skeletal age (SA), an indicator of maturity status at the time of observation; and third, to compare estimated ages at PHV reported for longitudinal samples of soccer players from Europe and Japan.

## MATERIALS AND METHODS

### Participants

Data for the present study were part of the *Coimbra Soccer Longitudinal Project*, which followed the guidelines established by the declaration of Helsinki [11]. Formal approval was obtained from the *University of Coimbra Sports Sciences and Physical Education Board*, and included agreements with the Presidents of the respective soccer clubs. Written consent was obtained from parents or legal guardians of the players, and players were informed that participation was voluntary and that they could withdraw from the study at any time.

The baseline sample included 87 U13 players 11–12 years of age from five clubs in the midlands of Portugal; the players were classified as *infantiles* in the Portuguese Soccer Federation. All players except one were of European ancestry. At baseline, the sample had 1–6 years of experience in soccer (median 3 years), and participated in 3–5 training sessions (~90 minutes) and one game (usually on Saturday) per week.

Heights and weights, among other anthropometric dimensions, were initially measured within a two week interval in December; players who persisted at the respective clubs were subsequently measured within the same two week interval in December over the next five seasons. All measurements were taken by a single observer (MJCS) at the University of Coimbra. Heights, with shoes removed, were measured to the nearest 0.1 cm using a stadiometer (Harpenden 98.603, Holtain Ltd, Croswell, UK). Weight was measured to the neared 0.1 kg using a SECA scale (model 770, Hanover, MD, US). Intra-observer technical errors of measurement were 0.27 cm for height and 0.47 kg for weight. Chronological age (CA) at each observation was calculated as the difference between date of birth and date of a hand-wrist radiograph (see below) for observations one, three and five, and between date of birth and date of measurement for observations two and four.

Over the five years, 59 players (68% of the baseline sample) had four or five annual height measurements. The longitudinal sample did not differ significantly from their 28 teammates at baseline: respectively, CA, 11.9 ± 0.5 and 11.7 ± 0.5 years; SA, 12.0 ± 1.4 and 11.8 ± 1.6 years; height, 144.8 ± 6.9 and 144.3 ± 6.5 cm; and weight, 37.6 ± 6.0 and 38.8 ± 7.0 kg; the distribution of players by pubic hair status also did not significantly differ.

### Parameters of the Adolescent Growth Spurt

The longitudinal height records of 58 players of European ancestry were successfully modeled with two methods to estimate parameters of the adolescent spurt: Superimposition by Translation and Rotation (SITAR) and Functional Principal Component Analysis (FPCA). The heights of one player of non-European ancestry limited to four observations were not successfully modeled.

The SITAR model [12, 13] available in the R package *sitar* [14] fits the raw height data for all players with a curve (defined as a Bspline), superposes the curves of all players, averages the curves and then back-projects the average curve into the original data as a growth model through uniform transformations: translation and rotation. A total of 269 measurements were available for the 58 players. Visual inspection of the model based on running plots with the raw data showed that the model fit the data very well. The mean residual was 0.0 cm by definition; the standard deviation of the residuals was 0.47 cm and the mean absolute value of the residuals was 0.36 cm.

The FPCA growth model [15] is based on a combination of general Functional Data Analysis (FDA) and FCPA [16, 17]. The complete postnatal growth curves of individual boys in the Brno Growth Study were the training set, which was fitted by the B-spline curves of the raw data for all soccer players. The splines were modeled with the FPCA procedure; 12 Principal Components (6 for phase and 6 for amplitude of the curves) were then used as a generative model to fit the newly analyzed data based on the Levenberg-Marquardt optimization algorithm. Details of the specific calculations and functions of the model are available in the R package *growthfd* [15, 18]. The mean of the 269 model residuals was 0.04 cm and the standard deviation of the residuals was 0.44 cm; the mean absolute value of the residuals was 0.33 cm.

The accuracy of estimates of parameters of the adolescent spurt in height depends on the model. Models differ significantly in the shape of the curve between points and in the extent to which they take into account information about the actual course of human growth, i.e., whether they are more ‘mathematical’ or more ‘empirical’. The present study computed estimates using two models that account for data of this nature. Both the SITAR and FCPA methods provided estimates of age, velocity of growth and height at TO and at PHV for each player.

Estimates of age at PHV based on one of the protocols (SITAR) in the present analysis were previously used in a study evaluating the validity of predicted ages at PHV among the soccer players [19]. The present study compares parameters of the growth spurt based on two different models, SITAR and FCPA. Of note, heights of one player in the earlier analysis were not successfully modeled with the two protocols used in the present study.

### Skeletal Age

Posterior-anterior radiographs of the left hand-wrist of players were taken at observations one, three and five. The Fels method [20] method was used to estimate SA. The mean difference between independent assessments of SAs of 20 radiographs by two individuals and the inter-observer technical error of measurement were, respectively, 0.03 ± 0.04 years and 0.12 years, while the inter-observer intra-class correlation was 0.99. Standard errors for SA assessments at observations one, three and five ranged, respectively, from 0.27 to 0.30 year (median 0.29), from 0.29 to 0.49 year (median 0.35), and from 0.30 to 0.48 (median 0.37) year.

### Analysis

Descriptive statistics (means and standard deviations) at each observation for the longitudinal sample were calculated for CA, height and weight, for SA and SA minus CA at the three observations, and for estimated ages at TO and PHV (years), velocities of growth at TO and at PHV (cm/year), and heights at TO and at PHV (cm) based on the SITAR and FPCA methods. The differences between parameters of the growth spurt with the two methods were evaluated with paired sample t-tests and tests of equivalence using 90% equivalence boundaries representative of a moderate effect (± 0.5 of Cohen’s d). Spearman rank order correlations (rho) between ages at PHV based on the SITAR and FCPA models and the differences of ages at PHV based on the respective models were calculated.

Each player was also classified as late (delayed), on time (average) or early (advanced) maturing based on ages at PHV with the two models and also on SAs at observations one, three and five. A band of plus/minus one standard deviation of the respective mean ages at PHV for the total sample defined on time or average maturity status. Estimates ages at PHV outside the range of plus/minus one standard deviation were classified as either late (age at PHV above one standard deviation of the respective mean ages) or early (age at PHV less than one standard deviation of the respective mean ages). Similarly, an SA within ± 1.0 year of CA defined average skeletal maturity status, while an SA younger than CA by > 1.0 year and an SA older than CA by > 1.0 year defined, respectively, late (delayed) and early (advanced) skeletal maturity status. The range of ± 1.0 year allows for error associated with assessments of SA and approximates standard deviations for SAs within specific CA groups [21]. Four players were skeletally mature at observation five and an SA was not assigned. At observation five, 42 players had radiographs, but three did not have height and weight measures; of the 40 players with measures of height and weight, one did not have a radiograph.

Means and standard deviations were calculated for ages at PHV for players in each of the maturity groups defined by the SITAR and FCPA methods, and also for SA-CA differences in the respective skeletal maturity status groups at observations one, three and five. Concordance of maturity status classifications based on the two estimates of age at PHV and on skeletal maturity status at the three observations was evaluated with chi square and unweighted Cohen’s Kappa coefficients.

In addition to estimated ages at PHV for the present sample of Portuguese players, ages at PHV for 12 longitudinal samples of soccer players from Europe and Japan were compiled from the literature [6]: six samples from Europe: Wales [22], Denmark [23], Belgium [24], Spain [25–27], England [28] and the Netherlands [29], and six samples from central Japan [30–34]. Estimates of age at PHV were based on a variety of methods, and several studies including the present study reported estimated ages at PHV based on two or three methods. Three studies provided estimates for subsamples of Spanish players from the same club. Excluding estimates based on graphic and incremental methods [22, 28] and the FPCA method (present study), and limiting the estimate for Spanish players to that based on the largest sample [27], ages at PHV in the 13 samples of soccer players from Europe and Japan were subjected to a meta-analysis using the methods available in the R-Package *metaphor* [35] within the R-software, version 3.5.3 [14]. Sample sizes, means and standard deviations for age at APHV in each of the 13 studies were used as estimates of effect size. The Random Effect Model was used as it can be reasonably assumed that the population with the same grand mean age at PHV was not sampled in the studies of soccer players (i.e., the populations actually differed in ages at PHV). The restricted maximum likelihood method (REML estimator) was used to estimate the between-sample variance (τ^2^, tau-squared).

## RESULTS

Descriptive statistics for CA, height and weight at each observation and for SA at observations one, three and five in the longitudinal sample of soccer players are summarized in Table 1. Corresponding statistics for parameters of TO and PHV, and results of t-tests and Cohen’s d are summarized in Table 2. Although quite similar, mean ages at TO, SITAR 11.2 ± 0.8 years and FPCA 11.0 ± 0.8 years, and mean ages at PHV, SITAR 13.6 ± 0.9 years and FCPA 13.7 ± 0.9 years, differ significantly. Estimated heights at TO, SITAR 141.1 ± 5.7 cm and FCPA 140.2 ± 6.0 cm, and at PHV, SITAR 157.1 ± 5.7 cm and FCPA 157.9 ± 5.6 cm, also differ significantly. In contrast, estimated velocities of growth in height at TO, SITAR 4.6 ± 0.5 cm/year and FCPA 4.6 ± 0.4 cm/year, and at PHV, SITAR 9.7 ± 1.3 cm/year and FCPA 9.8 ± 1.3 cm/year, do not differ significantly.

**TABLE 1:** Means (M) and standard deviations (SD) for chronological age (CA), skeletal age (SA), height and weight for the longitudinal sample of soccer players by observation (Obs).

Obs	N	CA, yrs		SA, yrs		Height, cm		Weight, kg	

		M	SD	M	SD	M	SD	M	SD
1	58	11.9	0.5	12.0	1.4	144.9	6.9	37.6	6.0
2	58	12.9	0.5			151.7	7.9	42.3	7.3
3	58	13.9	0.5	14.2	1.1	159.2	7.7	48.7	8.4
4	55	14.9	0.5			165.5	6.7	54.9	7.9
5^a^	40	15.9	0.5			169.3	5.3	60.1	6.3
5^b^	35	15.8	0.5	16.3	1.1	169.2	5.4	59.7	6.2
5^c^	4	16.8	0.2			171.5	5.0	64.9	6.1

a Total sample of players with measures of height and weight at observation five; one player did not have a radiograph;

b Not skeletally mature;

c Skeletally mature

**TABLE 2:** Means (M) and standard deviations (SD) for estimated parameters at take-off (TO) and at peak height velocity (PHV) based on the SITAR and FPCA methods, differences between the respective estimates (SITAR minus FPCA) with the two methods, and results of the t-tests.

	SITAR			FPCA			SITAR - FPCA			

Parameters	M	SD	Range	M	SD	Range	M	SD	t	Cohen’s d
Age at TO, yrs	11.24	0.79	9.94–13.00	10.99	0.82	8.55–12.81	0.25	0.70	2.73**	0.36
TO, cm/yr	4.62	0.52	3.37–6.06	4.58	0.36	3.85–5.67	0.03	0.47	0.56	0.07
Height at TO, cm	141.1	5.7	130.0–153.8	140.2	6.0	125.0–153.0	0.90	3.32	2.06*	0.27
Age at PHV, yrs	13.62	0.90	11.92–15.59	13.66	0.88	11.90–15.49	-0.04	0.12	2.60**	0.34
PHV, cm/yr	9.71	1.26	6.69–13.56	9.81	1.33	6.97–14.53	-0.10	0.87	3.80**	0.50
Height at PHV, cm	157.1	5.7	145.9–169.7	157.9	5.6	147.7–170.1	-0.51	1.02	0.84	0.11

* p < 0.05,

** p < 0.01

Estimated ages and heights at PHV with the two methods are highly correlated, 0.99 and 0.98 (p < 0.001), respectively, while the correlation for estimated PHVs with the two methods is slightly lower, 0.77 (p < 0.001). Correlations between estimated ages and heights at TO with the two methods, though lower, are significant, 0.62 and 0.84 (p < 0.001), while the correlation between estimated velocities of growth at TO with the two methods is lower 0.48 (p < 0.001).

The cross-tabulation of maturity classifications based on ages at PHV with each method is shown in Table 3. Overall, 90% of the players are classified as having the same maturity status based on SITAR and FCPA ages at PHV. Four of the six misclassified players have estimated ages at PHV close to the ± 1.0 cut-offs; the differences in ages at PHV (SITAR minus FPCA) are negligible, 0.08, 0.07, 0.05 and 0.08 year. The differences between ages at PHV for two players are somewhat larger, 0.35 and -0.35 year.

**TABLE 3:** Frequencies and cross-tabulations of maturity status classifications (late, on time, early)1 based on ages at PHV with the SITAR and FPCA models, percentage agreement, Chi square (*X^2^*) and Cohen’s Kappa (ĸ); means and standard deviations for ages at PHV in the respective maturity groups are also indicated.

Age at PHV: FPCA, yrs		Age at PHV: SITAR, yrs			Total

		Late	On time	Early	
		14.92 ± 0.35	13.54 ± 0.53	12.37 ± 0.20	

Late	14.98 ± 0.27	**8**	1	0	9
On Time	13.69 ± 0.52	3	**36**	0	39
Early	12.36 ± 0.26	0	2	**8**	10

	Total	11	39	8	58
		Agreement 90% *c*^2^ = 77.29* ĸ = 0.79*			

* (p < 0.01); ^1^On time (average) is an age at PHV within ± 1.0 year of the mean age at PHV for the total sample of 58 players with SITAR (13.62 ± 0.90 years): average, 12.72 to 14.52 years; late, > 14.52 years; and early, < 12.72 years; and with FPCA (13.66 ± 0.88 years): average, 12.78 to 14.54 years; late, > 14.54 years; and early, < 12.78 years.

Spearman correlations (rho) between maturity classifications based on the differences of SA minus CA and on age at PHV are moderate in early adolescence (~12 years, observation one), -0.53 (SITAR, p < 0.01) and -0.54 (FPCA, p < 0.001), and higher in mid-adolescence (~14 years, observation three), -0.77 with both methods (p < 0.001). The negative correlations indicate an earlier age at PHV among players with an SA in advance of CA (positive difference of SA minus CA).

Cross tabulations of maturity classifications (late, average or early) based on ages at PHV with SITAR and FPCA and on the difference of SA minus CA at observations one, three and five are summarized in Table 4. Maturity classifications based on ages at PHV and SA at observation one (~12 years) and three (~14 years) are concordant in, respectively, 59% and 71% of players for SITAR and in 62% and 74% of players for FPCA estimates. Kappa coefficients are relatively low at observation one, but moderate at observation three. Allowing for small numbers at observation five (~16 years), maturity classifications are concordant in 57% (SITAR) and 60% (FPCA) of the players, and the Kappa coefficient is moderate. Among the four skeletally mature players at observation five, two are classified as on time and two as early maturing based on ages at PHV. Mean ages at PHV for the four skeletally mature players are similar with SITAR (12.57 ± 0.38 years) and FPCA (12.65 ± 0.37 years), and are earlier than mean ages at PHV among non-skeletally mature early maturing CA peers, SITAR (12.90 ± 0.65 years) and FPCA (12.94 ± 0.66 years), respectively.

**TABLE 4:** Frequencies and cross-tabulations of maturity status classifications based on ages at PHV with the SITAR and FPCA models and on Fels skeletal ages (SA – CA) at observations 1, 3 and 5, and percentage agreement, Chi square (*c*^2^) and Cohen’s Kappa (ĸ), and means and standard deviations for SA – CA differences in the respective maturity groups are also indicated.

Skeletal Maturity Groups	Skeletal Age^2^	Maturity Groups				

		Age at PHV SITAR^1^				Age at PHV FPCA^1^			

		Late	On time	Early	Total	Late	On time	Early	Total
Observation 1									
Late	(-1.98 ± 0.98 yrs)	**3**	6	0	9	**3**	6	0	9
On Time	(-0.02 ± 0.59 yrs)	6	**25**	2	33	4	**26**	3	33
Early	(1.76 ± 0.62 yrs)	2	8	**6**	16	2	7	**7**	16
Total	(0.16 ± 1.38 yrs)	11	39	8	58	9	39	10	58
		Agreement 59%, *c*^2^ = 11.60**, ĸ = 0.25* Agreement 62%, *c*^2^ = 13.49**, ĸ = 0.31*							

Observation 3									
Late	(-1.48 ± 0.33 yrs)	**5**	3	0	8	**4**	4	0	8
On Time	(0.18 ± 0.46 yrs)	5	**29**	1	35	4	**30**	1	35
Early	(1.66 ± 0.54 yrs)	1	7	**7**	15	1	5	**9**	15
Total	(0.33 ± 1.07 yrs)	11	39	8	58	9	39	10	58
		Agreement 71%, *c*^2^ = 28.75*, ĸ = 0.45* Agreement 74%, *c*^2^ = 33.45**, ĸ = 0.51*							

Observation 5									
Late	(-1.76 ± 0.38 yrs)	**5**	0	0	5	**3**	2	0	5
On Time	(0.00 ± 0.72 yrs)	2	**14**	0	16	1	**15**	0	16
Early	(1.71 ± 0.41 yrs)	0	12	**5**	17	0	10	**7**	17
Mature		0	2	2	4	0	2	2	4
Total	(0.53 ± 1.33 yrs)	7	28	7	42	4	29	9	42
		Agreement 57%, *c*^2^ = 36.90*, ĸ = 0.45* Agreement 60%, *c*^2^ = 27.17*, ĸ = 0.37*							

* (p < 0.01);

1 See Table 3 for ages at PHV in the respective maturity groups;

2 On time – SA within ± 1.0 year of CA; late – SA behind CA by > 1.0 year; early – SA advance of CA by > 1.0 year

Ages at PHV of soccer players in the 13 longitudinal series are summarized in Table 5. Mean ages at PHV based on different methods of estimation within several samples are not different. The mean ages at PHV for Portuguese soccer players based on SITAR and FPCA are within the range of mean ages at PHV in the six longitudinal samples of soccer players in Europe, 12.9 to 14.2 years. The earliest estimated mean age at PHV, 12.9 years (standard deviation not reported), based on a two-level polynomial method, is for a sample of 33 Spanish players [25]; estimates based on the SITAR model for larger samples from the same club (n = 110 and 124) are later, 13.4 ± 0.8 years [26] and 13.5 ± 0.9 years [27], respectively.

**TABLE 5:** Ages at PHV (years) and PHV (cm/year) in 13 longitudinal samples of male soccer players in Europe (including the present study) and Japan.

	Competitive	APHV yrs	PHV cm/yr							

Country	Study	Method*	Level	Observations (obs)	N	M	SD	Range, yrs	M	SD
**EUROPE**										

Portugal	Present study	Sitar FPCA	prof club	11–12/15–16 yrs, 4–5 annual obs, 2003–2008	58	13.6	0.9	11.9–15.6	9.7	1.3
					58	13.7	0.9	11.9–15.5	9.8	1.3

Wales	Bell [22]	Graphic Moving incr Polynomials	school	12–15 yrs, 4 annual obs, 1981–1984	32	14.2	0.8		9.6	1.8
						14.1	0.8		9.3	1.5
						14.2	0.9		9.5	1.5

Denmark	Froberg et al. [23]	PB 1	local club semi-annual obs over 6 yrs, 1980s	11–16 yrs,	8	14.2	0.9	12.6–15.7		

Belgium	Philippaerts et al. [24]	Polynomials	prof club	10–13/14–17yrs, annual obs 1996–2000	33	13.8	0.8	12.3–15.9		

Spain (same club)	Carvalho et al. [25]	Polynomials	prof club	10–16 yrs, 4 obs 2009–2014	33	12.9		11.8–15.5†	8.1	

	Monasterio et al. [26, 27]	Sitar	prof club	10–11 yrs-16–18 yrs, > 10 obs, 2000–2020	110	13.4	0.8		9.9	1.8
					124	13.5	0.9		10.1	2.0

England	Parr et al. [28]	Sitar Graphic	prof club	5 seasons 12.4 ± 0.6 yrs baseline, 17–20 obs, 2013–2017	27	14.1	0.8	12.6–15.5	9.8	
					27	14.2	0.8			

Netherlands	Teunissen et al. [29]	PB 1	prof club	4 seasons 11.9 ± 0.8 yrs baseline, 16–25 obs, 2008–2012	17	13.8	0.7	12.6–15.2		

**JAPAN**										

Fukui Prefecture	Nariyama et al. [30]	PB 1	school	school records, 1970–1987, 6–18 yrs	83	13.7	1.1		8.8	1.1

Saitama	Saeki et al. [31]	Auxal	school	school records 7–12 yrs + obs JHS	88	13.3	0.9			

Tokyo	Takei et al. [32]	Auxal	rec league 6 obs over 2 yrs 2011–2016	school records +	201	13.4	0.9			

Shizouka	Chuman et al. [23]	Triple logistic	prof club	school record: sub-elite 7–12yrs, club elite obs at 13 yrs	48	12.9	0.9			
					16	12.6	1.0			

Shizouka	Chuman et al. [34]	Triple logistic	prof club	school records, club 6 obs, 7–15 yrs, 2008–2010	29	12.9	1.0			

* Methods: Moving inc (moving increments), PB 1 (Preece Baines model 1), Auxal (refers to the software package used; the authors do not specify which of the three models implemented in the Auxal program was specifically used to estimate ages at PHV);

† estimated 95% credible interval; prof club (professional club level); rec league (recreational league)

Estimated mean ages at PHV for players from professional clubs in Europe are somewhat earlier than those for players from a school and local club (Table 5). With the exception of the one subsample of players in Spain [25], means ages at PHV for club soccer players in Europe tend to be later than estimates for players at a professional club in Japan, 12.6 to 12.9 years, which are earlier than estimates for school and recreational league players in Japan.

**FIG. 1:**
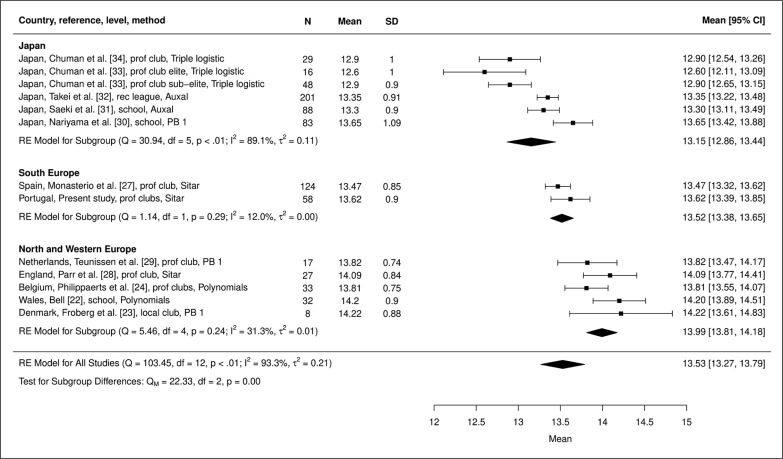
Aggregation of ages of PHV (years) in samples of male soccer players based on meta-analysis, including the subgroup analysis. Q: Cochran’s Q-statistic (weighted sum of squares), QM: Cochran’s Q-statistic for subgroups, I^2^: percentage of variability in effect sizes which is not due sampling error, τ2: between-study variance in a given set of samples (years squared); plots: means and 95% CIs of individual studies; diamonds: width represents 95% CI for each model aggregated by subsample and for all studies (below).

Results of the meta-analysis of ages at PHV in the 13 samples of soccer players from Europe and Japan indicate significant heterogeneity (Q [df, 12]) = 103.45, p < 0.001), while I^2^ for the model of all studies is relatively high (93.3%). The effect of geographic location (Japan and Southern, Northern and Western Europe) as a moderator of age at PHV was then evaluated. The Mixed Effect Model indicates a statistically significant moderator effect (Q_M_ [df, 2] = 22.33, p < 0.001); ages at PHV differ significantly among the geographic groups (Figure 1). Age at PHV is latest for players from Northern and Western Europe, earlier for players from Southern Europe, and earliest for players from Japan. Heterogeneity among samples in Northern and Western Europe (professional and local clubs and schools combined) is not significant (Q [df, 4] = 5.46, p = 0.243), while heterogeneity among the samples of professional clubs in Europe (Southern, Northern and Western Europe together) is significant (Q [df, 4] = 15.4260, p = 0.004). By inference, geographic distribution appears to be a more significant factor than level of competition, though the relatively small sample sizes in Northern and Western Europe may have reduced the statistical significance of differences among samples.

## DISCUSSION

Differences in estimated mean ages at TO and PHV and estimated mean PHVs based on the SITAR and FPCA models in the sample of 58 Portuguese soccer players, though statistically significant, were small in practical terms (Table 2). Estimated mean ages at TO (11.2 and 11.0 years) and at PHV (13.6 and 13.7 years) with, respectively, the SITAR and FPCA models among soccer players were also within the ranges of reported mean ages in longitudinal samples of European boys spanning the 1970s through the present: ages at TO, 10.4 to 11.8 years (21 estimates), and ages at PHV, 13.0 to 14.5 years (64 estimates). Mean PHVs among soccer players (9.7 and 9.8 cm/year) were also within the range of estimates in the general population, 7.8 to 11.5 cm/year (27 estimates) [6, the reference includes citations for the specific studies].

Concordance of maturity status classifications (late, average or early) based on ages at PHV and on SA minus CA was modest at initial observation, 11.9 ± 0.5 years and higher at the third observation, 13.9 ± 0.5 years (Table 4). The observations were consistent with relationships among indicators of maturity timing close to the time of PHV among 111 boys in the Wrocław Growth Study [36]. Correlations between estimated CAs at attaining SAs of 12.0 and 14.0 years and age at PHV were, respectively, 0.42 and 0.81. Correlations between the two estimates of age at PHV and the difference of SA minus CA in the 58 soccer players were similar at observations one (-0.54) and three (-0.77), i.e., advanced skeletal maturity status at 12 and 14 years was related to an earlier age at PHV, and the association was stronger closer to the time of PHV.

Estimated mean ages at PHV for the 58 Portuguese soccer players based on the SITAR and FPCA models were within the range of mean ages at PHV estimated with several different methods in six longitudinal samples of soccer players in Europe (Table 5). Though limited to a relatively small number of studies, results of the systematic analysis of the 13 samples of soccer players suggested earlier ages at PHV among players in Southern compared to Northern and Western Europe, and earlier ages at PHV among Japanese club players compared to European players (Figure 1). The trend towards earlier ages at PHV among Japanese compared to European soccer players was consistent with that noted in the general population of youth in both regions [6].

Variation in ages at PHV *among individual players* also merits attention; estimated ages at PHV (Table 5) were within the range of longitudinal samples of European boys, 11.3 to 17.3 years [6, 37, 38]. The relatively late CAs at initial observation and limited duration of several studies of soccer players may have affected the estimated ranges. In contrast, studies in Japan are unique in that they commonly use serial height records of players measured annually in April at their respective schools beginning at 7 years of age [30]; the school records were complemented by measurements taken at several leagues and clubs.

Variation in ages at TO among Portuguese players (Table 2) was in the range of ages at TO in three longitudinal samples of European boys, 9.0 to 15.0 years [39–41]. This variation has implications for monitoring the growth status of youth players as implemented by the English Premier League [9]. Heights and weights of all registered academy players 9 years and older are measured every three to four months. Along with corresponding observations for fitness and an academy-wide injury audit, the data provide a potentially unique opportunity to better understand the impact of the interval of the adolescent spurt upon fitness and performance and also on the incidence and burden of injury. Note, however, height measurements at such relatively close intervals require attention to inter- and intra-examiner measurement variability and also to diurnal and seasonal variation in growth. Moreover, heights should not be measured after training and scrimmages.

Based on monthly measurements of heights and weights of soccer players 11–19 years during the course of a season (September through April), estimated monthly increments of > 0.6 cm/month in height and of > 0.3 kg/m^2^/month in the BMI, and an estimated monthly decline of > 0.4 kg/m^2^/month in the BMI were associated with an increased risk of injury [42]. Extending the monthly increments in height through a year, it was suggested that an estimated velocity of growth in height ≥ 7.2 cm/year was indicative that a player was in his growth spurt [10, 42]. The range of estimated PHVs in the sample of 58 soccer players (Table 2), however, suggested that some players with rates of growth < 7.2 cm/year were in their growth spurts.

Epidemiological data suggest enhanced susceptibility to injury during the interval of the growth spurt, especially conditions associated with rapid growth, i.e., Osgood-Schlatter and Sever’s disease [43, 44], and overuse [45]. Use of developmentally appropriate training protocols (activities emphasizing core strength, balance, coordination, mobility, and limiting accelerations and decelerations) and management of training loads may serve to mitigate injury risk during the interval of rapid growth [46]. Some athletes may also experience temporary disruptions or regressions in motor performances during the interval of the growth spurt, commonly labeled as adolescent awkwardness [6]. Of potential relevance, recent evidence suggests that coach evaluations of match performances of youth soccer players tend to decline during the growth spurt, but return to prespurt levels at the cessation of the growth spurt [47]. When evaluating youth athletes, it thus is essential that coaches and others involved are aware of the individuality of growth and maturation during the interval of adolescence, specifically variation in timing and tempo of the spurt. Accommodating individual differences may include, for example, delaying decisions until after the growth spurt, reviewing player performance metrics prior to the onset and during the spurt, and/or allowing a player to play down an age group while they adjust to changes associated with the adolescent spurt [48].

Ages at PHV derived from longitudinal height records of individual youth should not be confused with estimates based on predicted maturity offset defined as the time before PHV, and predicted age at PHV estimated as CA minus predicted maturity offset [49, 50].

Though increasingly used in studies of youth athletes spanning preadolescence through adolescence, predicted estimates increase with CA and height at prediction and systematically differ from ages at PHV observed in longitudinal studies [6, 37–39, 51].

The present study is not without limitations. The sample was measured on only five occasions spanning 11 and 16 years. Although the CA range was consistent with other longitudinal studies of European soccer players, the selectivity of the sport (many youth are excluded prior to 11 years) and subsequent selectivity and/or differential drop-out as players pass through adolescence merits attention. Of relevance, the relatively late CA at initial observation and limited duration of the study may have influenced estimated parameters in some players. Estimates of skeletal maturity status were also not available at all observations which limited comparisons of skeletal maturity status and timing of PHV. And, the 13 longitudinal studies of soccer players used a variety of methods to estimate ages at PHV which may have influenced the meta-analysis. Although meta-analysis has several limitations, it is an effective means of aggregating data from studies of soccer players that have used different analytical protocols.

## CONCLUSIONS

Mean ages at PHV among Portuguese soccer players with SITAR and FCPA were, respectively, 13.6 ± 0.9 and 13.7 ± 0.9 years, and within the range of means and standard deviations for ages at PHV in the general population and among soccer players in Europe. Concordance of maturity status classifications based on age at PHV and SA (SA minus CA) was moderate at observation 1 (early adolescence) and strongest at observation 3 (~14 years, close to PHV). Analysis of ages at PHV in 13 longitudinal samples of European and Japanese soccer players suggested a geographic/ethnic gradient: northern Europe > southern Europe > Japan.

## Data Availability

The data that support the findings of this study are not publicly available due to departmental policy and privacy commitments to the study participants. Nevertheless, the data may be available upon reasonable request to Professor Manuel J. Coelho-e-Silva, Faculty of Sports Science and Physical Education, University of Coimbra, Portugal.
